# Role of pH on Nanostructured SERS Active Substrates for Detection of Organic Dyes

**DOI:** 10.3390/molecules26082360

**Published:** 2021-04-19

**Authors:** Viviana Mollica Nardo, Vincenzo Renda, Sebastiano Trusso, Rosina Celeste Ponterio

**Affiliations:** 1 IPCF-CNR, Istituto per i Processi Chimico-Fisici, V.le F.S. d’Alcontres 37, 98158 Messina, Italy; trusso@ipcf.cnr.it (S.T.); ponterio@ipcf.cnr.it (R.C.P.); 2 Arpa Sicilia UOC Area Mare, Lungomare Cristoforo Colombo 4521, Loc. Addaura, 90149 Palermo, Italy; vrenda@arpa.sicilia.it

**Keywords:** SERS-substrates, pH, organic-dyes, PLD

## Abstract

Surface Enhanced Raman Spectroscopy is commonly used as analytical improvement to conventional Raman spectroscopy, able to respond to qualitative diagnostic enquiries, which involve low-concentrated molecular species in complex matrix. In this paper, we described fabrication, characterization and testing of a type of SERS-active substrates realized specifically to detect pigments in work of art. In particular, we detailed the SERS activity of nanostructured noble metal films deposited by pulsed laser ablation onto glass and polishing sheets substrates. The SERS response of the substrates was tested against the presence of some organic dyes in aqueous solutions. Measurements were performed at different pH values, in acidic or basic range, in order to investigate its role in the adsorption mechanism, thus fostering the SERS amplification. In addition, we checked the possible deterioration of the structural properties of the substrates that could occur in presence of alkaline or acidic environment. SERS activity of the substrates was tested against a commonly dye used as a SERS standard (Blue Methylene). Thereafter, substrates have been tested on two organic dyes (Alizarine red-S and Brazilwood), which had proven to be Raman active but present also either a weak Raman scattering cross section and/or a high fluorescence emission. The substrates have proven effective in amplifying Raman scattering of all dyes, quenching troubling fluorescence effects. Furthermore, they have proven to be stable in the pH range between 3 and 11. Furthermore, we carry out of vibrational DFT-calculation of dyes that provide a complete description of the observed SERS spectra.

## 1. Introduction

Surface-Enhanced Raman Scattering (SERS) effect consists in a large amplification of the normal Raman signal occurring when a molecule is adsorbed on a nanostructured surface [1,2,3]. The amplification of the Raman scattering signal is attributed either to the electromagnetic (EM) [4] mechanism or to a chemical based mechanism [5]. The latter is based on the charge transfer between the absorbed molecules and the metallic surface while the former is based on the strong amplification of the external EM field near corrugated metallic nanostructured surfaces where surface plasmon resonances occur. In such a case, the amplification of the Raman signal depends on the metal; silver provides the most intense SERS amplification followed by gold and copper. Due to its sensitivity, down to single molecule detection, chemical specificity and label-free nature, many applications, ranging from surface chemistry, biomedical, environmental and security sensing, are now SERS-based [6,7]. Actually, vibrational spectroscopies like infrared and Raman are widely used in the cultural heritage field, being a non-destructive technique able to detect pigments in work of art. However, Raman signal of pigments, usually of organic origin, is hindered by intense fluorescence emissions. Nevertheless, when molecules are adsorbed on a metallic surface, fluorescence is quenched, and this, together with the presence of the SERS effect, leads to the chance of acquiring well defined Raman, a combination that opened the way to the use of SERS spectroscopy in the cultural heritage field [8,9,10,11].

Depending on the nature of the substrate, SERS measurements can be performed following different procedures. In case of colloidal noble metal nanoparticles (NP), obtained for example by chemical methods [3] or by pulsed laser ablation in liquid environment (PLAL), measurements are performed by mixing the dye in solution with NPs colloidal suspension. In case of nanostructured noble metal thin films deposited onto rigid support, like glass or c-Si, the latter can be soaked into the dyes solution and then extracted and left to dry; alternatively, some drops of the dye solution can be casted onto the substrates. Nevertheless, regardless the SERS active substrate nature, molecular adsorption mechanism on the metallic surface is of primary importance in order to exploit Raman signal enhancement to the best. Usually, molecules interact with the metallic surface via weak long-range forces that determine the molecule affinity toward a particular metal. Other parameters can affect the adsorption kinetics like temperature, metallic surface charge state, and, in case of solutions, ionic strength and pH value. Concerning organic dyes, several studies on the effect of pH variation on the SERS intensity were reported in literature [12,13,14,15]. In these studies, colloidal silver or gold NPs, obtained by chemical methods, were employed. Usually, when adopting photochemical routes for the growth of SERS, active NPs residual chemicals are present in the final colloidal solution that can give rise to unwanted Raman signals or modify the adsorption mechanism of the molecules on the metallic surface. In previous works, we grow gold and silver nanostructured thin films onto glass or c-Si slides by pulsed laser ablation (PLA) in a vacuum chamber in presence of a controlled Ar atmosphere. Different surface morphologies, ranging from isolated NPs and metallic island to percolated thin films, were observed as a function of some critical deposition parameters, i.e., Ar pressure, target to substrate distance and deposition time. The technique, being the deposition carried out at room temperature and in presence of an inert gas atmosphere, has the advantage to be intrinsically clean and with virtually no limitations on the nature of the substrate. The films showed peculiar SERS activity, the SERS amplification factor depending on the surface morphology [16,17,18,19,20]; optimized substrates were used to detect and characterize organic dyes of interest in the field of cultural heritage [21,22,23]. Such characteristics can be fully exploited in the cultural heritage field only when pigments can be collected from the surface of a work of art, and it is quite clear that the sampling technique should be able to avoid or minimize unwanted damages to the surface. To this purpose, silver and gold NPs were deposited by means of PLA on the surface of commercial polishing sheets, the original use of which was the polishing of optical fibers [24,25,26]. Such substrates are able to collect small amount of pigments after being softly swept onto the surface of the sample [22,27]. Nevertheless, the adsorption of the collected material to the surface of the NPs is not fully guaranteed. In previous works, we observed that an acidic environment (pH = 2 using HCl) allowed the detection of the SERS spectra of aqueous solution of Perampanel (an antiepileptic drug) at the concentration level of 10–5 M, suggesting that the charge state of the molecule plays a relevant role in the SERS measurements [28,29]. In particular pH, acidic or alkaline depending on the chemical nature of the analyte, can play a role in the adsorption mechanism, thus fostering the SERS amplification.

**Figure 1:**
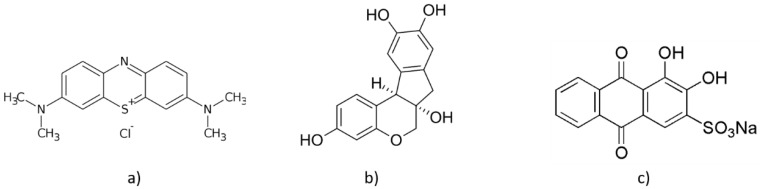
Molecular structure of the dyes: (**a**) methylene blue; (**b**) brazilwood; (**c**) alizarin red-S.

In this work, we present a study of the SERS activity of nanostructured films deposited by PLA onto glass and polishing sheets as a function of pH values. The aim is to check if a deterioration of the structural properties of the substrates occurs in presence of alkaline or acidic environment and test whether and how pH changes affect the SERS activity. To this purpose, two organic dyes, Alizarine red-S and Brazilwood, as well as a widely used SERS standard Methylene Blue (MB) (Figure 1), were dispersed in aqueous solutions in the pH range from 3 to 11. Finally, we combine Surface Enhanced Raman Spectroscopy (SERS) experiments with density functional theory (DFT) computations of the Raman frequencies of dyes.

## 2. Results and Discussion

As mentioned in Introduction Section, we analyzed three dyes with Ag and Au nanostructured films deposited by PLA onto glass and polishing sheets as a function of pH values. The dye molecules tested with both substrates are reported in Figure 1.

### 2.1. SERS on Metal Decorated Glass Slides

#### 2.1.1. Methylene Blue Dye

**Figure 2:**
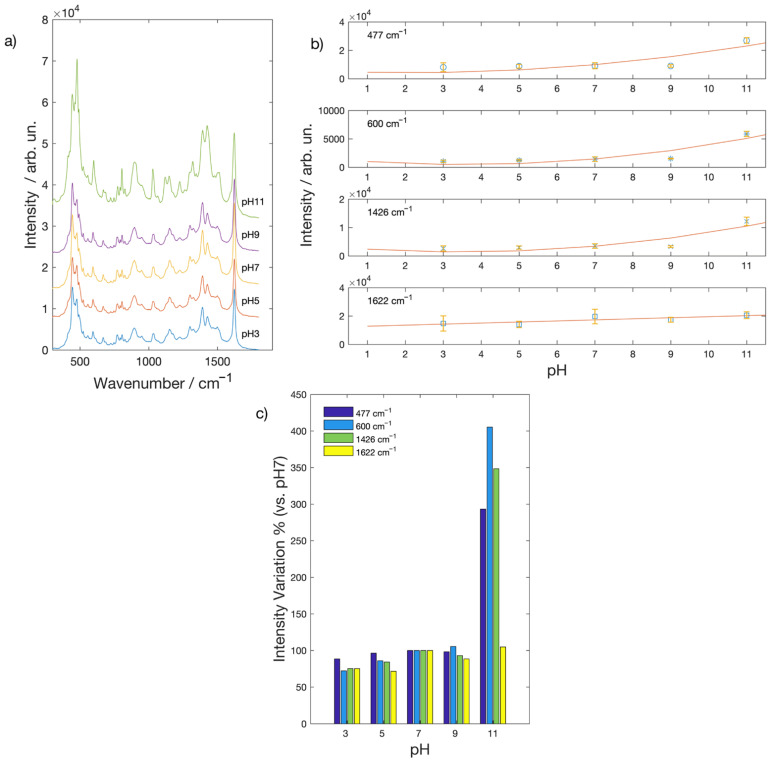
(**a**) SERS spectra of a MB 2.5 × 10^−4^ M solution at different pH values acquired on Ag SERS active substrates; (**b**) intensity behavior of some MB Raman peaks as function of the pH (lines are a guide for the eye); (**c**) intensity variation of Raman peaks in % with respect to the value at pH = 7.

The SERS spectra of a 2.5·10^−4^ M MB aqueous solution acquired on silver nanostructured film deposited onto glass slides were reported in Figure 2. Clear SERS spectra were obtained at any pH value. Characteristics peaks of MB, in fact, can be observed at 444 cm^−1^ [δ(C-N-C)], 477 cm^−1^ [δ(C-N-C)], 600 cm^−1^ [δ(C-S-C)], 1390 cm^−1^ [α (C-H)], 1426 cm^−1^ [ν_asym_(C-N)] and 1622 cm^−1^ [ν_ring_(C-C)] [27]. In order to assess how pH changes affect the SERS signal, we report in Figure 2b the intensity of some selected Raman peaks as a function of pH. Intensities are weakly affected in the pH range between 3 and 9, but some peaks showed a marked intensity increase at the pH value of 11. In Figure 2c, we report the intensity variation with respect to the value at pH = 7. While the intensity of the 1622 cm^−1^ Raman peak shows no or little variation as a function of pH, other peaks show a × 3–4 increase. Such a variation clearly points out a different chemical interaction between the substrate and the molecule as a consequence of protonation/deprotonation processes that can alter the molecular non-covalent bonding to the metallic film and are effective at pH = 11. For example, the increase of the 477 cm^−1^ peak with respect to the 444 cm^−1^ peak was attributed to the presence of MB monomers on the surface [30].

**Figure 3:**
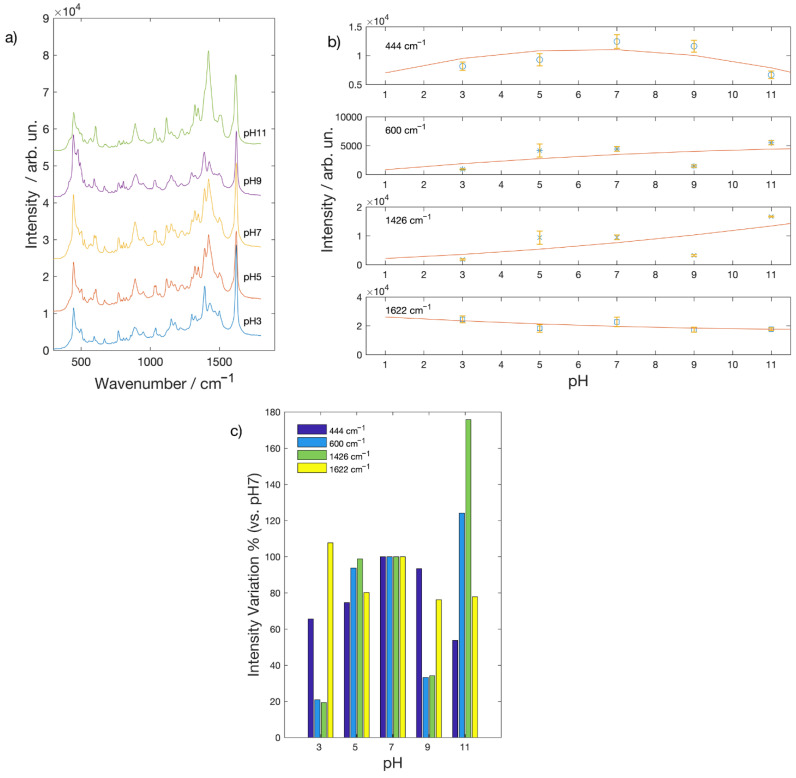
(**a**) SERS spectra of a MB 2.5 × 10^−4^ M solution at different pH values acquired on Au SERS active substrates; (**b**) intensity behavior of some MB Raman peaks as function of the pH (lines are a guide for the eye); (**c**) intensity variation of Raman peaks in % with respect to the value at pH = 7.

The situation is quite different for the spectra acquired on the surface of Au nanostructured substrate. As it can be seen in Figure 3a, also in this case, SERS spectra can be obtained at each pH value. Nevertheless, some features are at difference with respect to the spectra acquired on the silver substrate. In particular, the relative intensities of some Raman peaks and their behavior as a function of pH. The intensity of the peaks in the low frequency portion of the SERS spectra are, in fact, lower with respect to the peak at 1622 cm^−1^ contrary to what was observed in the spectra of MB acquired on the Ag substrate (see Figure 2a). It is well known that the nature of the substrate plays a role depending on the affinity of a molecule to a given metal so that changing the pH can improve or even allow the adsorption of a molecule on the surface where it can be detected. In Figure 3b is reported the intensity of some Raman peak as function of pH. The peak at 477 cm^−1^ is now very weak and little dependent on pH, so that in this case, we reported the intensity of the peak at 444 cm^−1^. Peak’s intensity behavior as a function of pH, as it can be seen in Figure 3c, is less clear with respect to the one observed for the silver substrate. The peak at 1622 cm^−1^ is still the one that show the lower dependency on pH; all the other peaks show a decrease in intensity with respect to the neutral condition, both in the alkaline and the basic pH range, with the exception of peaks at 600 and 1426 cm^−1^ that show a marked increase at pH = 11. The very low intensity of the peak at 477 cm^−1^ attributed, as reported above, to the presence of monomer on the silver surface, is a clear indication that MB show a very different affinity towards silver and gold; such an occurrence should be taken into consideration when detection and identification of organic dyes is performed with silver or gold SERS active substrates.

This finding shows how the affinity of a molecule with a metallic surface change as function of the nature of the metal but also how it is possible to modify the adsorption by changing the pH value and to improve the SERS intensity.

#### 2.1.2. Brazilwood and Alizarin Red-S Dyes

**Figure 4:**
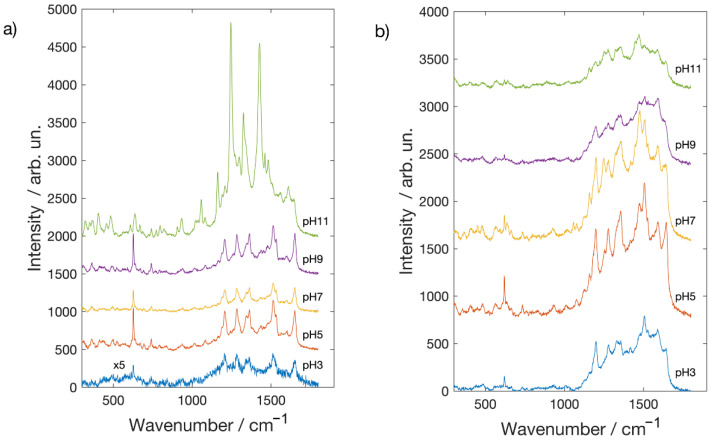
SERS spectra of Alizarin Red S on glass slides coated with (**a**) Ag NPs and (**b**) Au NPs.

**Figure 5:**
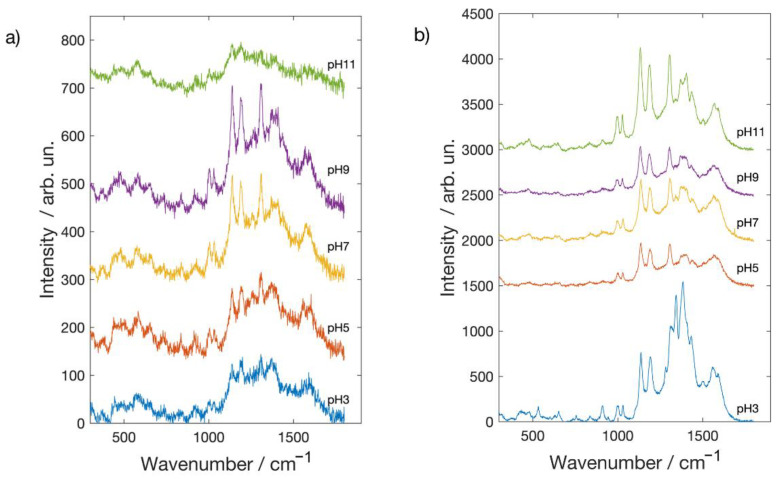
SERS spectra of Brazilwood on glass slides coated with (**a**) Ag NPs and (**b**) Au NPs.

In Figure 4 and Figure 5 are reported the spectra of Alizarin red-S and Brazilwood, acquired both on silver and gold covered glass slides at different pH values. Considering Alizarin red-S SERS spectra [31,32], acquired on the Ag NPs covered substrate (see Figure 4a), at pH 3, very weak features are discernible, while clear spectra were obtained at pH values between 5 and 11. SERS spectrum obtained at pH = 11 shows the highest intensity together with a different profile. Intense Raman peaks, in fact, appear at 1428 [ν(C-C)_arom_], 1327 [ν(C-C)], 1246 [ν(C-O)], 1162 cm^−1^ being the signature of deprotonation processes of the two hydroxylic groups that occur at alkaline pH [30]. It is worth to mention that such mechanism is not effective in the presence of the Au nanostructured surface. Looking at Figure 4b, in fact, weaker and noisier spectra are recorded on the Au NPs covered glass slide pointing out how, as it was observed for MB, Alizarin red-S shows a markedly different affinity towards silver and gold [18,19]. Concerning the pH dependence, spectra are little dependent on pH in the acidic range, being the spectra very similar to the one collected in neutral condition. Contrary to what was observed in the case of silver covered substrate, under basic conditions, quality of the spectra deteriorates, and moreover, there is no evidence of the spectral changes observed at pH 11 on silver.

Brazilwood [33,34] derived from Caesalpinia, whose main constituent is brazilin, but brazilein is also present. The structural formulae of brazilin and brazilein are depicted in Figure S3. It is interesting to note that brazilin is the major constituent in the crude dye, and brazilein can be isolated in large quantities when the dye is exposed to air and light. Concerning brazilwood dye, it is worth to mention that, despite it not being possible to obtain a normal Raman spectrum either in a high concentrated solution or in the powder form, clear bands are visible in the SERS spectra (Figure 5) at 1305 [v(C-O) + δ(OCC) + δ(CH2), 1187 and 1133 [δ(CCH) + v(C-C)], 1028 [ν_ring_], 1000 cm^−1^ [v(C-C) + v(C-O)] [27]. When comparing SERS spectra obtained on silver with the gold ones, Ag NPs mildly amplify Raman signals, while Au NP enhancement is much more marked. The behavior as a function of pH is different as well. On the silver substrate, the best Raman spectra are obtained in the pH range 5 and 7 while at the limits of pH range (3 and 11), tested in this work, brazilwood spectral features are very weak and noisy. In the spectra recorded on the Au covered substrates, all the characteristics of brazilwood Raman peaks are clearly observable at each pH value.

### 2.2. SERS on Metal Decorated Polishing Sheets

Metal decorated polishing sheets are ad hoc fabricated SERS substrates able to quench the dyes fluorescence and to enhance the Raman signal in Cultural Heritage filed. The substrates can be gently swabbed, for example, on a painted surface or on small decorated fragments in order to retain small quantities of the superficial layer. Raman spectra, then, are collected on the substrate surfaces avoiding in such away direct laser exposition of the work of art.

**Figure 6:**
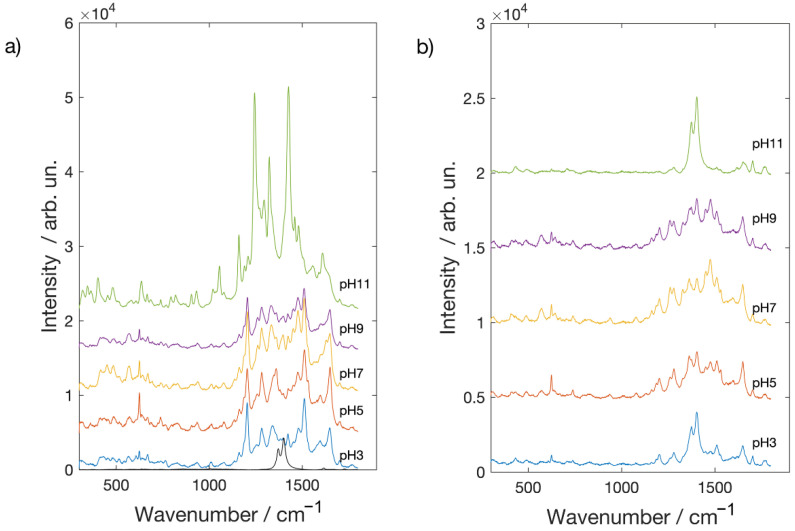
SERS spectra of Alizarin red-S acquired on polishing sheets coated with (**a**) Ag NPs and (**b**) Au NPs.

**Figure 7:**
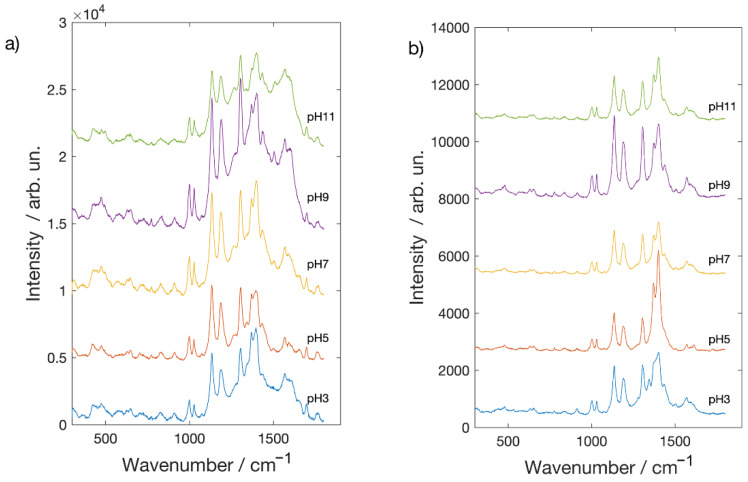
SERS spectra of Brazilwood acquired on polishing sheets coated with (**a**) Ag NPs and (**b**) Au NPs.

**Figure 8:**
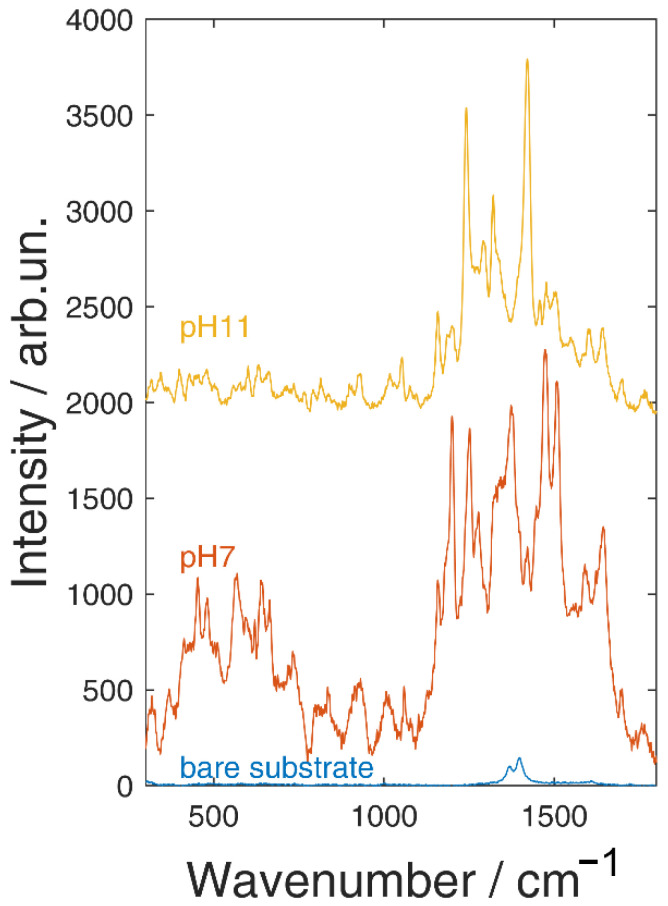
SERS spectra of Alizarine red-S collected by an Ag NPs covered polishing sheets and then diluted by drops of pH 7 and pH 11 solutions.

In Figure 6 and Figure 7 are reported the SERS spectra of Alizarin red-S and Brazilwood acquired on the surface of the polishing sheets covered by silver and gold NPs. Spectrum at the bottom of Figure 6a corresponds to surface of the polishing sheet as it was registered before the deposition process. It is characterized by the presence of two peaks at about 1370 and 1400 cm^−1^ due to the aluminum oxide powder glued to the substrate. Concerning Alizarin, red-S adsorbed on the silver covered substrate SERS amplification is observed for any value of pH. The behavior as function of the pH value is nearly identical to what observed in the case of the glass substrate, including the spectral modifications at pH 11, as they were registered on the glass substrate. Similar results are observed in case of the gold covered substrate (Figure 6b), even if with a higher enhancement with respect to the glass. Features observed in the pH 3 and pH 11 spectra are from the substrate (compare with the spectrum at the bottom of Figure 6a). Brazilwood SERS spectra on polishing sheets coated with metal nanoparticles present the same signature bands observed on glass substrates. It can be easily noticed, comparing results obtained on silver covered substrate with the gold covered one, that the enhancement is much more marked on the silver substrate. This behavior is opposite to that observed in the case of the glass substrates. Such a finding can be understood if a better affinity exists, not between the molecule and the metal but between the molecule and the aluminum oxide surface. Nevertheless, summarizing the results, it is quite clear that each molecule shows a peculiar affinity toward the gold and silver nanostructured surface where the SERS mechanism is affective. The choice of the SERS active substrate then, is of particular relevance when detection and identification of organic pigment should be performed. Changes in the pH values can help in improving adsorption and hence the Raman signal, but at the same time, they can induce spectral changes that have to be considered in the identification procedure. While pH changes can be easily obtained when measurements are performed on dyes in solution, the situation is different when they are removed by a surface using the polishing sheets. In this case, dyes are in the form of tiny particles on the surface of the polishing sheets. They can still be identified through SERS measurements [21], but adsorption can be improved in this case by adding on the surface, after the removal procedure, some drops of an aqueous solution with a given pH. SERS spectra are then acquired after evaporation of the solvent. As a proof of concept in Figure 8 are shown the SERS spectra of dye after a mild sweep procedure on an Alizarin red-S covered surface. Then, some drops of aqueous solution at pH 7 (neutral) and pH 11 were added. The results are nearly identical (including the spectral features changes at pH 11) to what observed in case of measurements performed after soaking of the substrate in the corresponding solutions. Such a finding indicates that it is possible to drive the adsorption of the dye after the removal procedure.

### 2.3. DFT Calculation Results

As pointed out in the Methods section, the dyes have been considered for the Raman analyses carried out by means of quantum-mechanical computational approaches.

In Supplementary Materials Tables S1–S3, we reported the band wavenumbers of SERS spectra of dyes in comparison with DFT results. Table S1 reports band wavenumbers of methylene blue at pH 7 in comparison with literature spectra [30] and DFT-calculate spectrum and their tentative assignments. The isolated MB was previously optimized at B3YLP/6-311++G level of theory, followed by Raman spectra calculations. Thanks to the simulation of the isolated dye molecule, it is possible to reproduce all vibrational bands. There is a good agreement between the observed SERS features and the calculated spectrum. Differences in the positions of most of the peaks are within 10 cm^−1^, and the largest difference is observed for the peak 1347 and 1622 cm^−1^.

Table S2 reports the comparison between SERS spectrum of brazilwood dye with brazilin and brazilein by reference and DFT-simulation. Thanks to DFT calculation, we can assume that are present both formulae of brazilin and brazilein; experimental SERS contain peak at 776 cm^−1^ attributed to brazilin and 656 cm^−1^ imputable to brazilein [35]. In spectra range between 1000–1700 cm^−1^, experimental and theoretical spectra are in good agreement.

Table S3 includes SERS spectrum of Alizarin red-S in comparison with DFT-simulation of deprotonate structures. In particular, we compare SERS obtained at pH7 with monoanionic-structure and SERS obtained at pH 11 with anionic ([−2; −3)] structures. All isolated molecules have been optimized at the computational 6-311++G, and Raman frequencies have been evaluated by using the same theory level. Experimental SERS spectrum at pH 7 is in good agreement with DFT frequencies of isolate alizarin red-S while SERS at pH 11 is in good agreement with alizarin −3. Some discrepancies are related to the presence of peaks in the SERS spectrum, at pH 11, in comparison with the theoretical ones, i.e., the peaks at 934 and 1645 cm^−1^, probably imputable to the interaction with metal surface.

## 3. Materials and Methods

### 3.1. Chemicals and Reagents

All chemical reagents (dyes, acids and bases) were purchased from commercial sources (Sigma–Aldrich, St. Louis, MO, USA and Zecchi, Italy). Methylene blue aqueous solution was prepared at the concentration level of 2.5 × 10^−4^ M solutions while Alizarin red-S and Brazilwood aqueous were prepared at the concentration level of 10^−2^ M. Solutions have been prepared at pH values of 3, 5, 7, 9 and 11, using diluted solutions of HCl and NaOH added dropwise. A pH-meter (Hanna Instruments HI 9025C, Padova, Italy) was used to control pH changes after each addition of titrant. SERS measurements were performed after soaking the substrates in the solutions for 1 h and left them to dry in air.

### 3.2. Pulsed Laser Deposition

Pulsed Laser Deposition (PLD) was performed using a homemade experimental setup. The physics underlying the formation of Ag and Au nanostructured thin films by PLD can be found in previous works [16,17,18,19,20]. Ablation was performed with a KrF excimer laser (Compex 205, Lambda Physik, Coherent Inc., Santa Clara, CA, USA) that provides light pulses at the wavelength of 248 nm with a pulse time width of 25 ns. The energy of the laser pulses was adjusted using an external optical attenuator together with a regulation on the laser voltage [25]. The laser fluence at the target surface was of about 2.0–3.0 J/cm^2^, the deposition was halted after 25,000 laser pulses. Ag and Au targets were positioned on a rotating holder, in order to minimize the surface damage, positioned at a distance of 35 mm far from the substrate holder. Deposition takes place in presence of a controlled Ar atmosphere that favors aggregation among atoms leading to the formation of NPs that land on the substrate with very low residual kinetic energies. Thin films were deposited onto two types of substrates: flexible polishing sheets coated with an aluminum oxide powder, with a rms roughness of 0.3 μm, and glass slides. Deposited under such conditions the surface morphology of the films is characterized by the presence of metallic island resulting from the coalescence processes among NPs on the substrate surface. Silver substrates should be used as soon as possible after the deposition; their SERS activity, in fact, is observed to decrease within weeks when left in the air; on the contrary, gold substrates are more stable and can be used even within months without any appreciable degradation of the SERS response. The Raman enhancement factor (EF) is defined as the ratio between the observed SERS activity (I_SERS_), measured on Ag and Au covered glass substrates, and the normal Raman activity (I_R_), recorded onto uncovered glass substrate. EF was evaluated comparing the intensities of the C-C stretching peaks at 1618 cm^−1^ of MB at 10^−4^ M concentration adsorbed on the nanostructured Ag and Au surfaces with respect to MB at 10^−2^ M concentration adsorbed onto an uncovered glass substrate. The resulting EF were about 7 × 10^3^ and 1.6 × 10^4^ for Ag and Au covered substrates, respectively.

### 3.3. Raman Spectroscopy Measurements

Raman spectra were acquired using an HR800 micro-Raman spectrometer (Horiba Jobin-Yvon, Northampton, UK). Spectra were excited by the 632.8 nm He-Ne laser line, employing a 600-lines grating and a 50× long working distance objective. Laser energy density and integration times were chosen in order to maximize signal-to-noise ratio and to avoid sample degradation. Baseline subtraction was performed using the airPLS routine (adaptive iteratively reweighted penalized least squares) [36]). All the spectra were normalized with respect to the acquisition times and the laser energy at the sample surface.

### 3.4. UV–VIS Absorption Spectroscopy

UV–VIS absorption spectra of Ag and Au nanostructured thin films deposited on glass slides by PLA were collected using a homemade setup (Avantes AvaLight-DHS Deuterium-Halogen Light Source coupled with the Avaspec 2048L spectrometer, Apeldoorn, The Netherlands) in the range 300–1000 nm.

The LSP bands of Ag and Au NPs are positioned at maxima at 483 and 703 nm respectively. The LSP positions are shifted with respect to the one observed for isolated NPs, pointing for a detuning of the optical transitions due to dipole-dipole interaction among nearby nanostructures as a consequence of clustering process among NPs [22,30].

### 3.5. DFT Calculations

All calculations have been performed with the Gaussian 09 [37] program using the B3LYP hybrid functional. First, geometry optimizations in gas phase have been performed using 6-311++G atomic basis set for all the atoms. In the second step, at the same level, vibrational calculations were performed [38]. Molecular geometries pictures were realized using the GaussView5.0 graphical software. The computed vibrational frequencies were scaled—in a standard way—by a factor of 0.98 to yield a better agreement with the experimental vibrational frequencies Computational studies were performed in order to assign all the vibration frequencies of the Raman spectra of dyes.

## 4. Conclusions

In conclusion, we tested the SERS response of some organic dyes of interest in the cultural heritage field. SERS spectra were acquired on different kinds of substrate, namely, glass slides and polishing sheets covered by nanostructured silver and gold thin films deposited by the PLD technique. The role played by the acidic/alkaline environment was investigated in the pH range between 3 and 11. It was observed that besides the different affinity of the dyes against silver and gold, the SERS activity was dependent on the pH. The behavior was different depending on the considered dye. Marked differences with respect to normal condition (pH 7) were observed at pH 3 and 11, depending on the dye and on the metal. A simple procedure is proposed in order to improve the SERS response in the case of sample collection by the swabbing procedure exploiting the SERS signal increase fostered by the by the acidic/alkaline environment.

## Data Availability

The data presented in this study are available in article and supplementary material.

## Supplementary Materials

The following are available online, Table S1: Band wavenumbers of SERS spectrum of methylene blue at pH 7 in comparison with literature [30] spectra and DFT-calculate spectrum and their tentative assignments. Table S2: Band wavenumbers of SERS spectrum of brazilwood dye at pH 7 in comparison with brazilin and brazilein [32] and DFT-calculate spectra and their tentative assignments. Table S3: Band wavenumbers of SERS spectrum of Alizarin red-S dye at pH 7and pH 11; comparison with DFT-calculate spectra of deprotonated molecules and their tentative assignments. Figure S1: SERS spectrum of methylene blue at pH7 in comparison with DFT-calculate spectrum. Figure S2: Comparison of the Raman spectra of Methylene-Blue at pH7 recorded on Ag, Au covered and uncovered glass, substrates. The calculated EF values are reported. Figure S3: Structural formulae of a) brazilin and b) brazilein and comparison between SERS spectrum at pH7 and DFT-calculate spectra. Figure S4: Structural formulae of a) alizarin red S [-1] b) alizarin red S [-2] and c) alizarin red S [-3] and comparison between sers-spectra at pH 7 and 11 with DFT-calculate spectra of anionic species.

## Sample Availability

Not available.
