# Supplementary material for: Role of pH on Nanostructured SERS Active Substrates for Detection of Organic Dyes
Source: Molecules. 2021 Apr 19;26(8):2360. doi: 10.3390/molecules26082360 (PMC8073486; doi:10.3390/molecules26082360)

Table S1: Band wavenumbers of SERS spectrum of methylene blue at pH 7 in comparison with literature [30] spectra and DFT-calculate spectrum and their tentative assignments.

**Solid MB [26]**	**MB on Ag nanocaps [26]**	**Our SERS-Ag**	**Calculate Raman isolated MB**	**Assignment**
445	444	444	439	δ(C-N-C)
		477		δ(C-N-C)
497	502		493	
	615	600	596	δ(C-S-C)
677	684	668	672	γ(CH)
768		772	761	
	890	886	884	
		952	953	
	1032	1039		β(CH)
1067	1070		1063	
	1119	1118	1111	γ(CH)
		1151	1152	
1181		1178	1186	υ(CN)
	1205	1224	1234	
1272	1265		1282	
	1307	1298	1310	
1331		1323	1344	
		1347	1365	
1396		1390	1399	α(CH)
1441	1420	1426	1440	ν_{asym}(CN)
			1476	
		1500	1495	
1554	1563		1550	ν_{asym}(CC)
1618	1613	1622	1646	ν_{ring}(CC)

υ, stretching; α, in plane ring deformation; β in-plane bending; γ out-of plane bending; δ, skeletal deformation

Table S2: Band wavenumbers of SERS spectrum of brazilwood dye at pH 7 in comparison with brazilin and brazilein [32] and DFT-calculate spectra and their tentative assignments.

**Brazilin [32]**	**Brazilein [32]**	**SERS-Au**	**Calculate Raman isolated Brazilin**	**Calculate Raman isolated Brazilein**	**Assignment**
423	442		439	439	
473		476	462	470	
490	492				
501					
	531		517	525	
549	547		564	540	
642	641		642	620	
		656		658	
687			689		
732	731		736	721	
			752		
767	765	776	776		γ(CO)/γ(CH)
792			792		
			835	846	
		916	909	917	
945					
990		1000	987	995	v(CC)/ v(CO)
1032	1031	1028	1050		*δ*(CH)
		1133	1113	1113	*δ*(CH)
1172	1169	1187	1183	1176	*δ*(CH)/*δ*(CC)
1230	1214		1223	1230	v(CO)/v(CC)
1260			1285	1286	
1320	1320	1305	1300	1324	v(C-O) + δ(OCC) + δ(CH_2)
	1365		1340	1372	
1451	1437	1435	1481	1419	
1525				1520	
	1564			1575	v (C=C)
			1591		v (CC)
1614	1612		1622	1622	
	1697			1646	
2858	2850				
2900					
2940	2938		2963	2971	
3060	3062		3057	3065	

υ, stretching; α, in plane ring deformation; β in-plane bending; γ out-of plane bending; δ, skeletal deformation

Table S3: Band wavenumbers of SERS spectrum of Alizarin red-S dye at pH 7and pH11; comparison with DFT-calculate spectra of deprotonated molecules and their tentative assignments.

**Alizarin-S [31]**	**SERS-Ag pH7**	**SERS-Ag pH11**	**Calculate Raman isolated Alizarin-S**	**Calculate Raman Alizarin-S [-2]**	**Calculate Raman Alizarin-S [-3]**	**Assignment**
409	406		415			γ(CH)/ γring
		460	462	466	459	δ(OH)/δ(C=O)/ δring
	491	481	501	500	500	
566	569		580	589	570	γ(OH)/γ(CH)
						δring/δ(OH)
	623	608	627			
		636	642	637	624	δ(CH)/ δring
648	645			651	651	δ(CH)
				672	665	
	742	740	744	720	761	δring/δ(OH)
		795		775		γ (CH)/ γ (CO)
			815	830		
903		906		900	900	γ(CH)
935	937	934				
			1011	1008	994	υ (CC)/ δ (CCC)
					1022	
1068	1078	1058	1050	1042	1056	δ (CCC)
		1083	1105			ν (SO_3) sym
					1111	
				1152		
1160	1161	1163	1176	1166	1173	ν(SO_3) asym
1203	1202	1209	1200	1200		ν(CC)/δ(CH)/δ(CCC)
1235		1245	1230	1248	1236	
1265	1260				1262	δ(OH)/δ(CH)
1289	1279		1270	1282		ν(C=O)
		1300	1293	1310	1303	ν(C=O)
1330	1327	1325	1333		1330	ν(CC)
1350	1361			1385	1378	δ(CO)
	1402				1406	
1441	1449	1429	1427	1447		δ(CH)
1460	1476	1463		1474	1468	ν(C=O)/ν(CC)/δ(CH)
		1482	1480	1488		
				1516	1502	
	1511		1536		1536	
1590				1586		
		1611	1600	1605		ν(CC)
1634	1650	1645				ν(C=O)

υ, stretching; α, in plane ring deformation; β in-plane bending;
γ out-of plane bending; δ, skeletal deformation

Figure S1: SERS spectrum of methylene blue at pH7 in comparison with DFT-calculate spectrum.

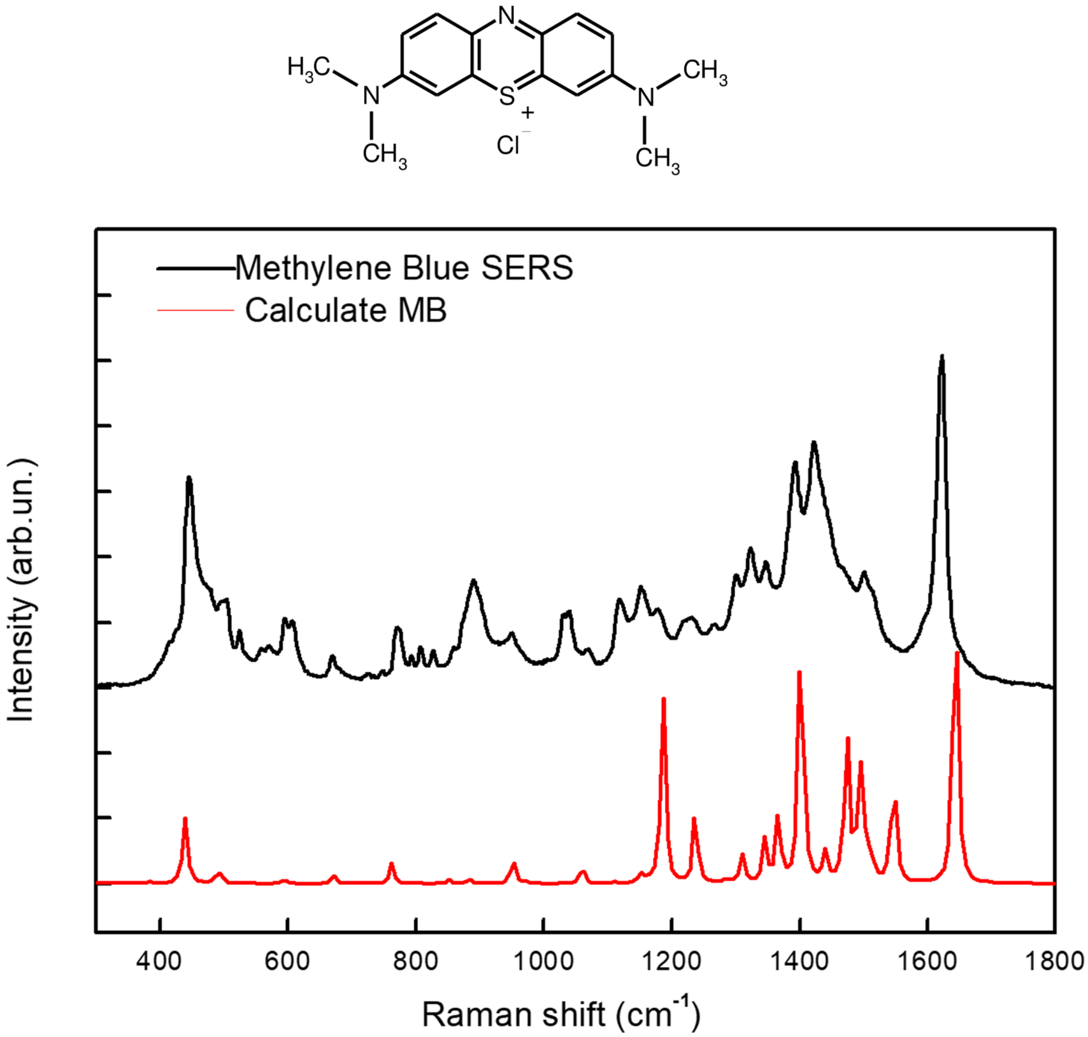

Figure S2: Comparison of the Raman spectra of Methylene-Blue at pH7 recorded on Ag, Au covered and uncovered glass, substrates. The calculated EF values are reported.

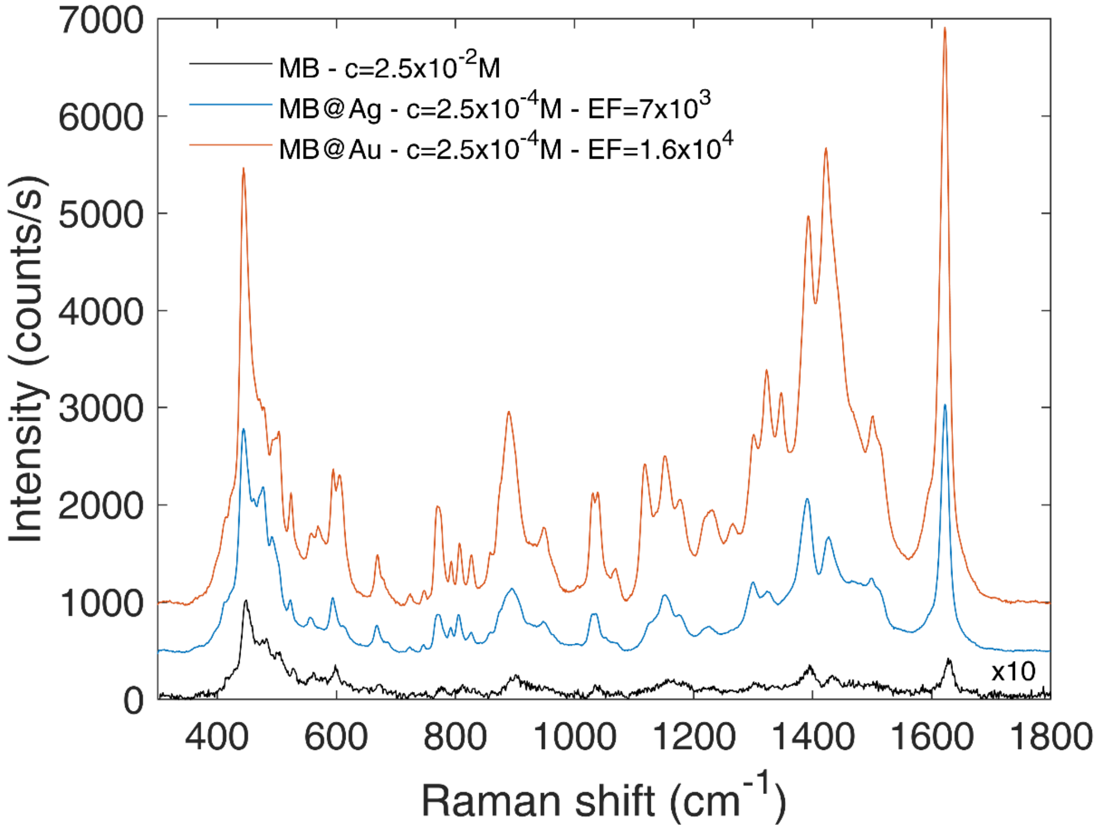

In Fig. SI2 we report a comparison of the Raman spectra of Methylene-Blue at pH7 recorded an uncovered glass substrate and Ag and Au covered ones. The EF was estimated using aqueous solution of methylene blue (MB), a dye used as a standard for the EF quantification. A MB 2.5x 10^{-2} M solution was used to in order to have a clear signal on the uncovered substrate, while a $2.5x10^{-5}$ M solution was used on the Ag and Au covered substrates. We used the intensity of the C-C Raman stretching peak at 1618 cm^{-1} after spectra normalization with respect to integration times and optical density filter values adopted for the measurements. The resulting EF were about $7x10^{3}$ and $1.6x10^{4}$ for Ag and Au covered substrates respectively.

Figure S3: Structural formulae of a) brazilin and b) brazilein and comparison between SERS spectrum at pH7 and DFT-calculate spectra.

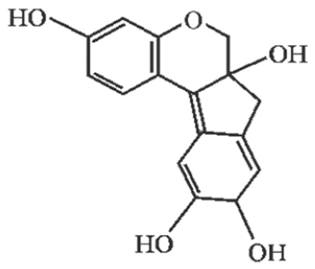

a) brazilin

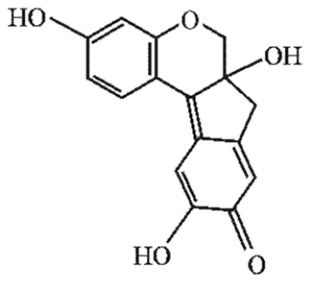

b) brazilein

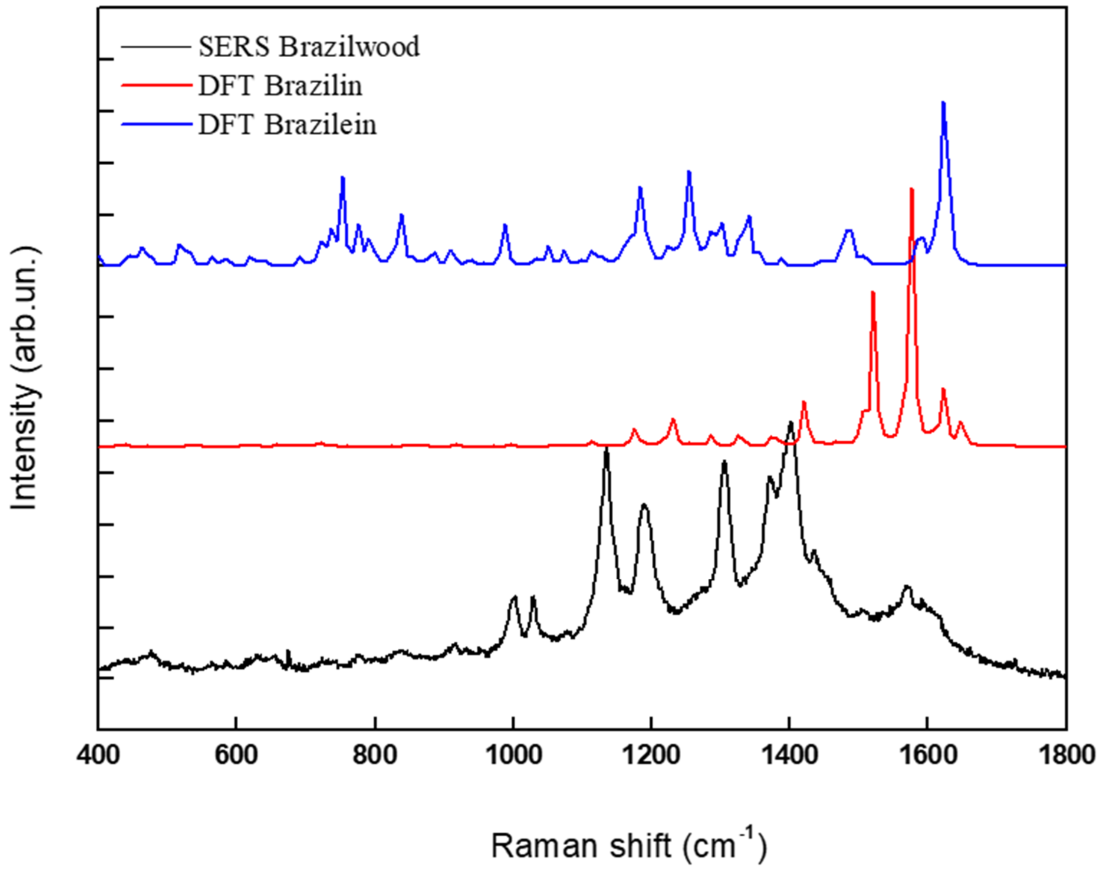

Figure S4: Structural formulae of a) alizarin red S [-1] b) alizarin red S [-2] and c) alizarin red S [-3] and comparison between sers-spectra at ph 7 and 11 with DFT-calculate spectra of anionic species.

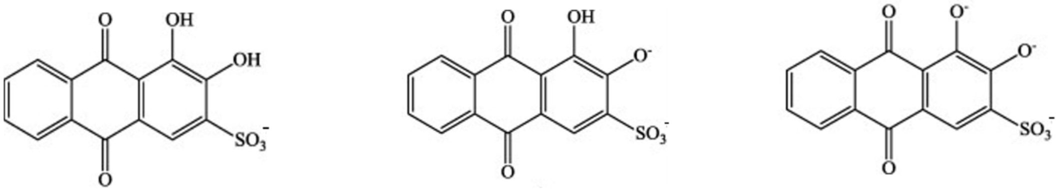

a) b) c)

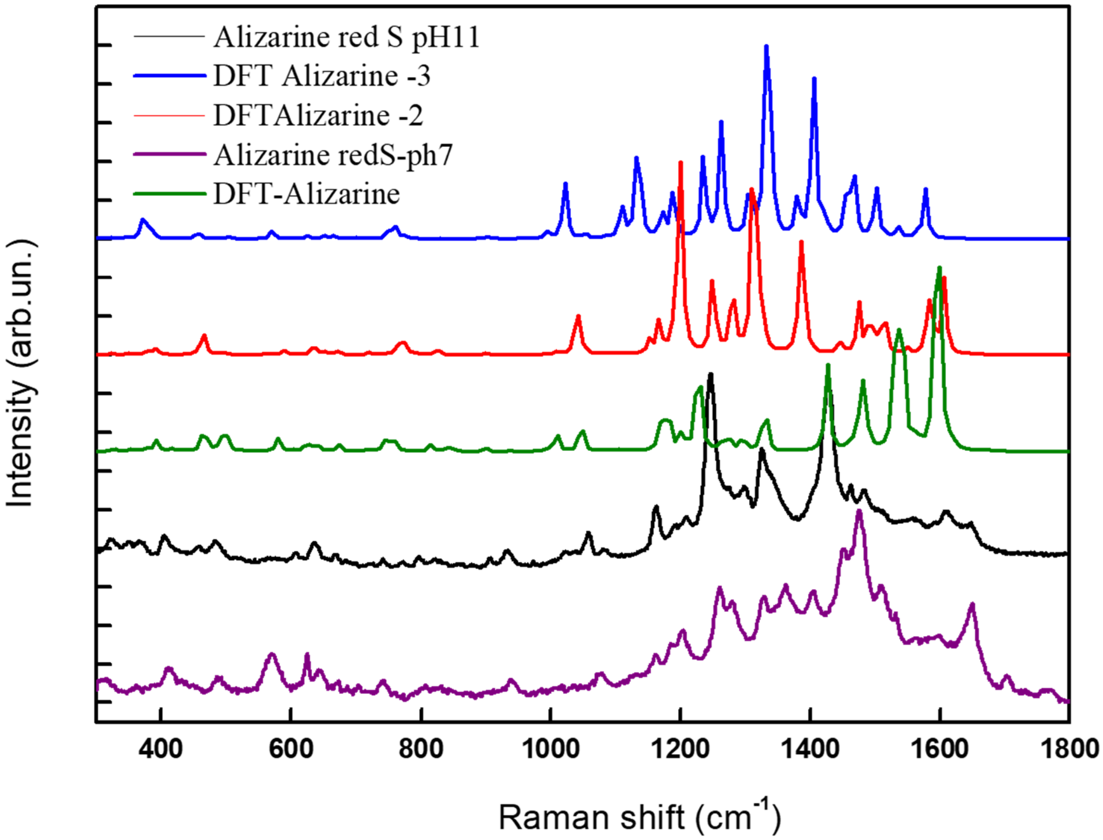

Supplement: Supplementary file 1 [file molecules-26-02360-s001.zip › molecules-1179528-supplementary.pdf]
